# Engineering the Morphology and Properties of MoS_2_ Films Through Gaseous Precursor-Induced Vacancy Defect Control

**DOI:** 10.3390/nano15221723

**Published:** 2025-11-14

**Authors:** James Abraham, Nigel D. Shepherd, Chris Littler, A. J. Syllaios, Usha Philipose

**Affiliations:** 1 Department of Physics, University of North Texas, 1155 Union Cir., Denton, TX 76203, USA; 2 Department of Material Science and Engineering, University of North Texas Discovery Park, 3940 N Elm St., Denton, TX 76207, USA; 3 Center for Microelectronics in Extreme Environments, University of North Texas Discovery Park, 3940 N Elm St., Denton, TX 76207, USA

**Keywords:** MoS_2_, CVD growth, vacancy defects, charge transport

## Abstract

The morphology, structure, and composition of CVD-grown molybdenum disulfide (${\mathrm{MoS}}_{2}$) films were investigated under varying precursor vapor pressures. Increasing sulfur vapor pressure transformed the film morphology from well-defined triangular domains to structures dominated by sulfur-terminated zigzag edges. These morphological changes were accompanied by notable variations in both structural and electrical properties. Non-uniform precursor vapor distribution promoted the formation of intrinsic point defects. To elucidate this behavior, a thermodynamic model was developed to link growth parameters to native defect formation. The analysis considered molybdenum and sulfur vacancies in both neutral and charged states, with equilibrium concentrations determined from the corresponding defect formation reactions. Sulfur vapor pressure emerged as the dominant factor controlling defect concentrations. The model validated experimental observations, with films grown under optimum and sulfur-rich conditions, yielding a carrier concentration of $9.6\times 10^{11}$ ${\mathrm{cm}}^{-2}$ and $7.5\times 10^{11}$ ${\mathrm{cm}}^{-2}$, respectively. The major difference in the field-effect transistor (FET) performance of devices fabricated under these two conditions was the degradation of the field-effect mobility and the current switching ratio. The degradation observed is attributed to increased carrier scattering at charged vacancy defect sites.

## 1. Introduction

To fully exploit the potential of two-dimensional transition metal dichalcogenides (2D-TMDs) in various applications, it is essential to understand and control their properties. Among the TMD family, molybdenum disulfide (${\mathrm{MoS}}_{2}$) has attracted significant interest due to its intriguing properties [1,2,3] including valley polarization. The breaking of the inversion symmetry and the spin–orbit coupling in monolayers of ${\mathrm{MoS}}_{2}$ lead to coupled spin and valley physics and give a new degree of freedom to carrier transport. This allows for new device concepts based on valleytronics and in spin–valley coupling.

The quasi-two-dimensional (2D) crystal of ${\mathrm{MoS}}_{2}$ consists of a single layer of molybdenum (Mo) atoms sandwiched between two layers of sulfur (S) atoms, all arranged in a close-packed hexagonal structure. The inherently layered nature of 2D-TMDs leads to pronounced anisotropy in its electrical, chemical, and thermal characteristics. A key feature of ${\mathrm{MoS}}_{2}$ is its bandgap transition from an indirect bandgap of ≈1.3 eV in its bulk form to a direct bandgap of ≈1.8 eV in its monolayer form. This transition makes ${\mathrm{MoS}}_{2}$ especially attractive for 2D optoelectronic applications [4,5]. Several of the unique properties of ${\mathrm{MoS}}_{2}$ arise from quantum confinement effects, which are partially attributed to the material’s dimensionality in relation to its Bohr radius. Moreover, the layered configuration of 2D-TMDs like ${\mathrm{MoS}}_{2}$ not only results in strong light–matter interactions but also supports relatively high charge carrier mobility. Several ${\mathrm{MoS}}_{2}$-based device concepts have been demonstrated, including field-effect transistors (FETs) with mobilities as high as 320 ${\mathrm{cm}}^{2}\;V^{-1}\;s^{-1}$, current on/off ratio of $10^{8}$ at room temperature [6], and inverters with gain up to 16 [7]. Other promising applications include phototransistors [8], photosensors [9], gas and biological sensors [10,11], and strain sensors [12].

One of the primary factors influencing the electronic and chemical behavior of these layered semiconducting materials is their defect density. To synthesize electronic grade ${\mathrm{MoS}}_{2}$ for practical applications, it is necessary to achieve high-quality film growth with large areal coverage. Various techniques have been used for the controlled growth of large-area ${\mathrm{MoS}}_{2}$ films, including mechanical exfoliation and chemical vapor deposition (CVD). The CVD synthesis of TMDCs of the form M$X_{2}$ (where M = Mo, W and X = S, Se) has attracted considerable attention owing to its ability to yield films with superior uniformity, high reproducibility, and precise thickness control [13,14]. The CVD growth of ${\mathrm{MoS}}_{2}$ has proven particularly challenging, mainly due to the non-uniform vapor pressure of the precursors, resulting in the growth of ${\mathrm{MoS}}_{2}$ monolayers with lower carrier mobility compared to exfoliated flakes [15]. This is attributed to the high temperature growth process that results in the formation of various defects, including point defects in the film [16]. The ${\mathrm{MoS}}_{2}$ films grown by CVD mostly exist in the 2H (hexagonal) phase, which is believed to be the most thermodynamically stable phase [17]. The ${\mathrm{MoS}}_{2}$ domains can form in various shapes—such as triangles, hexagons, and three-pointed stars—attributed to variations in the Mo:S vapor flux ratio during the growth process [18]. Therefore, it is critical to understand the factors that govern the growth kinetics of ${\mathrm{MoS}}_{2}$ films as well as the evolution of their structural and point defects under different growth conditions. The novelty of this work lies in establishing a clear correlation between the growth parameters—specifically the precursor pressure ratios—and the resulting variations in defect concentration, morphology, as well as the optical and electrical properties of the ${\mathrm{MoS}}_{2}$ films.

The first part of this paper focuses on a systematic study of intrinsic point defect formations in CVD-grown monolayer ${\mathrm{MoS}}_{2}$ films. The defect concentrations are explored through their formation energies as a function of the S vapor pressure, over a wide range of experimental conditions, from Mo-rich to S-rich. CVD growth typically happens in conditions where there is either an excess or a deficit of S atoms, and so the defects under study are Mo and S vacancies and charged versions of these. Since vacancy defects—V_Mo_ and V_S_—have lower formation energies than antisite defects such as Mo_S2_ (a Mo atom substituting an $S_{2}$ site) and S2_Mo_ (an $S_{2}$ site substituting a Mo atom), the stability and evolution of these vacancies are the primary focus of this study. Sulfur vacancies (V_S_) do not exhibit a strong tendency to aggregate; consequently, sulfur di-vacancies (V_S2_) possess approximately twice the formation energy of mono-vacancies [16,19] and are, therefore, not considered in this work. Additionally, the influence of V_S_ and V_Mo_ on the n-type conductivity commonly observed in CVD-grown ${\mathrm{MoS}}_{2}$ is examined. The second part presents the growth dynamics of ${\mathrm{MoS}}_{2}$, specifically examining the effects of S concentration and growth pressure on the shape evolution of the crystal domains.

## 2. Materials and Methods

Monolayers of ${\mathrm{MoS}}_{2}$ crystals were synthesized by CVD growth following the sulphurization of molybdenum trioxide (${\mathrm{MoO}}_{3}$), a widely studied and perfected procedure [20,21,22]. The monolayers were grown on a Si/SiO_2_ substrate at 800 °C, using ${\mathrm{MoO}}_{3}$ and pure S as precursors. A continuous flow of argon (Ar) gas was used to stream the S vapors to the ${\mathrm{MoO}}_{3}$ coated substrate. The precursor mass, the inert gas flow rate, and the deposition substrate position were kept constant during the 10-minute growth process. The films were grown under varying S pressures by adjusting the mass ratios of the Mo:S precursors to range from 1:5 to 1:80. This control over the Mo:S ratio changes the S vapor pressures in the CVD growth chamber, resulting in shape variations in the ${\mathrm{MoS}}_{2}$ films, changing from a truncated triangle to triangular and, finally, becoming three- and six-point star-shaped structures. Optimal growth conditions were observed at an Mo:S ratio of 1:15, with the formation of triangular films.

The as-grown ${\mathrm{MoS}}_{2}$ crystals were subsequently transferred to an Si/SiO_2_ substrate using a modified polymethyl methacrylate (PMMA)-assisted technique. The simplified transfer procedure addresses the common challenges of flake distortion during transfer and poor adhesion. In this method, a 1 μm thick layer of PMMA diluted in chloroform is spin-coated onto the as-grown ${\mathrm{MoS}}_{2}$/SiO_2_ substrate and heated at 70 °C for 5 min. The PMMA-coated sample is then submerged in deionized (DI) water overnight, with the substrate edges trimmed to allow water penetration beneath the PMMA layer. The PMMA layer, along with the ${\mathrm{MoS}}_{2}$ flakes, is carefully transferred to a new substrate, ensuring that the layer remains flat throughout the process [23]. The concentration of PMMA in chloroform is critical; if the solution is too concentrated, polymer residue will remain on the film surface even after acetone treatment, whereas an overly diluted solution fails to lift off the ${\mathrm{MoS}}_{2}$ effectively. The substrate is then heated at 70 °C for another 2 min to promote adhesion between the PMMA/${\mathrm{MoS}}_{2}$ layer and the new substrate. To remove the polymer, the sample is submerged in dichloromethane for 1 min and then immersed in acetone at 50 °C for 20 min. This technique has been proven to minimize mechanical stress and preserve the structure of the transferred flakes.

**Figure 1:**
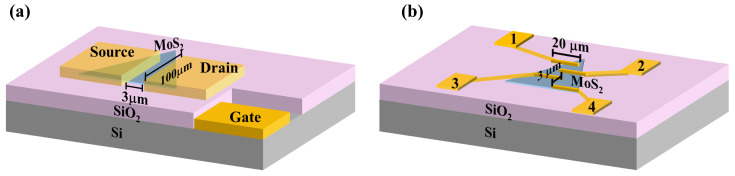
Schematic diagram of fabricated devices. (**a**) Gated FET structure (3 × 100 μm) constructed with monolayer ${\mathrm{MoS}}_{2}$ on a highly doped p-type Si substrate with a 285 nm thick ${\mathrm{SiO}}_{2}$ layer; (**b**) 4-terminal device (3 × 20 µm) for contact resistance measurements.

The morphology of the films were studied using a scanning electron microscope (SEM: SU1510, Hitachi High Technologies, Japan) and the monolayer step height determined using an atomic force microscope (AFM: Mobil S, Nanosurf AG, Switzerland). The composition was studied using Raman spectroscopy (inVia confocal Raman microscope: Renishaw PLC, UK). Electrical contacts were patterned on monolayer ${\mathrm{MoS}}_{2}$ films deposited on heavily p-type doped Si wafers capped with 285 nm thick ${\mathrm{SiO}}_{2}$. The devices were patterned using standard photolithography (MJB3 mask aligner: Karl Suss, Germany), followed by metal (Au) deposition to define two-terminal (3 × 100 μm) and four-terminal (3 × 20 μm) devices. A schematic of the two devices with contact dimensions and geometry is shown in Figure 1. Electrical measurements were performed using a semiconductor parameter analyzer (B1500A: Agilent Technologies, USA), with the device mounted in a temperature-controlled cryostat (Janis) equipped with a temperature controller (Lakeshore 332 controller: Lake Shore Cryotronics, USA).

## 3. Results

Sulfurization of ${\mathrm{MoO}}_{3}$ is a widely reported method for synthesizing ${\mathrm{MoS}}_{2}$ thin films. In this section, we first examine the evolution of vacancy defects in the as-grown films. This is followed by a discussion on the morphological evolution of ${\mathrm{MoS}}_{2}$ films grown at a constant temperature with varying Mo:S ratios. Finally, we compare the performance of ${\mathrm{MoS}}_{2}$ thin-film FETs fabricated from films grown under different S pressures.

### 3.1. Analysis of Vacancy Defect Formation in MoS_2_

The MoO_3_ sulfurization process is expressed by the following equation [24]:

(1) $$2\;{{\mathrm{MoO}}_{3}}_{\left(g\right)}+7\;S_{\left(g\right)}\to 2\;{{\mathrm{MoS}}_{2}}_{\left(s\right)}+3\;{{\mathrm{SO}}_{2}}_{\left(g\right)}$$

The growth of monolayer ${\mathrm{MoS}}_{2}$ involves the direct conversion of gaseous precursors into a solid product via a vapor–solid–solid (VSS) mechanism, driven by mass transfer across the gas–solid interface [25]. Growth imperfections—particularly due to non-uniform precursor vapor pressures—lead to the formation of intrinsic point defects, thermodynamically governed by the growth temperature (T) [26]. Each defect type can be described by a corresponding chemical reaction, typically accompanied by the generation of charge carriers. The resulting mass–action relations were formulated under equilibrium conditions. These intrinsic defects often manifest as vacancies, where atoms are absent from their lattice positions. The mass–action law, together with the charge neutrality condition, is used to calculate the defect concentrations as a function of the S partial pressure ($P_{S}^{7}$) during the growth phase. The vacancy concentrations were theoretically estimated using the following formula [27,28]:

(2) $$\left[V\right]=N_{\mathrm{sites}}N_{\mathrm{config}}\exp\left(\frac{S}{k}\right)\exp\left(\frac{-E_{f}}{kT}\right)$$

where *S* is the entropy of the defect and $E_{f}$ is the formation energy of the defect. The term $N_{sites}$ is the number of sites per unit volume in the lattice, and $N_{config}$ represents the number of possible defect configurations, assumed to be one for vacancy defects. Boltzmann’s constant and absolute temperatures are represented by *k* and *T*, respectively. The vacancy formation energy ($E_{f}$) is associated with Mo and S; both charged and neutral defects were obtained by ab-initio calculations using QuantumATK V-2023.12, using the generalized gradient approximation of Perdew, Burke, and Ernzerhof (PBE) for a 5 × 5 × 1 supercell [29,30]. The entropy contribution is challenging to compute accurately, since it requires large supercell phonon calculations and must account for vibrational, configurational, and electronic contributions. In this work, elemental entropy was estimated from standard thermochemical data corresponding to the CVD growth temperature, providing a physically reasonable approximation [31].

The S vapor pressure plays a crucial role in the growth of monolayer ${\mathrm{MoS}}_{2}$. The minimum S vapor pressure ${\left(P_{S}\right)}_{\min}$ required to favor the chemical reaction can be estimated using the following approximation:

(3) $${\left(P_{S}\right)}_{\min}^{7}=\left(\frac{K_{f}}{{\left(P_{{\mathrm{MoO}}_{3}}\right)}^{2}}\right)$$

The above equations were solved to determine the concentrations of various vacancies as a function of ${\left(P_{S}\right)}_{\min}$, revealing that the minimum S vapor pressure required for ${\mathrm{MoS}}_{2}$ growth is approximately $1.43\times 10^{-3}$ atm. The condition for electron neutrality is expressed as [32]

(4) $$n+\left[V_{Mo}^{-}\right]=p+\left[V_{S}^{+}\right]$$

**Figure 2:**
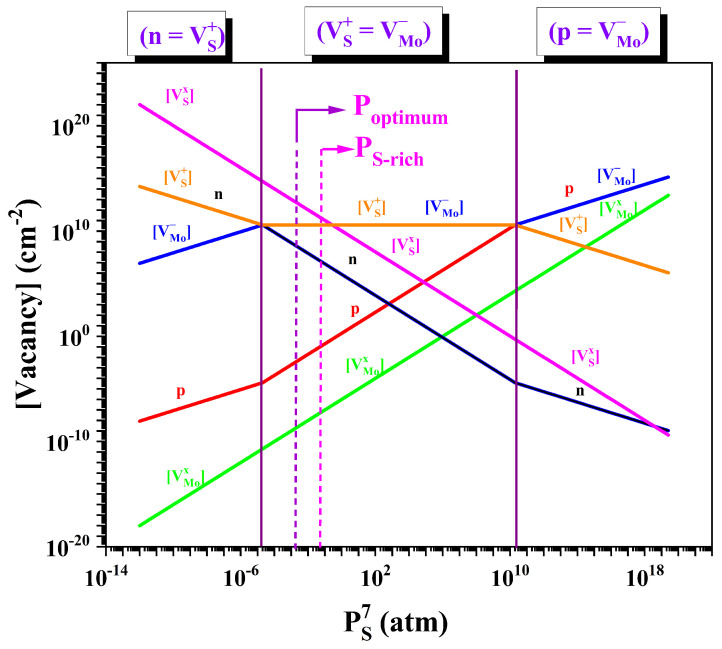
Vacancy defect concentration in ${\mathrm{MoS}}_{2}$ film, determined as a function of S vapor pressure.

The superscripts “+” and “−” denote positively and negatively charged species, respectively, while the superscript “x” indicates a neutral species. Based on the electron neutrality approximation, three distinct growth regimes are defined: $n=[V_{S}^{+}]$, $\left[V_{Mo}^{-}\right]=\left[V_{S}^{+}\right]$, and $p=[V_{Mo}^{-}]$. The concentrations of free electrons and holes are denoted by *n* and *p*, respectively, while the free electrons and holes appearing in the reaction equations are represented as $e^{-}$ and $h^{+}$. The equations in Table 1 are modified to calculate the concentrations of the defects $\left[V_{S}^{+}\right]$, $\left[V_{S}^{x}\right]$, $\left[V_{\mathrm{Mo}}^{-}\right]$, and $\left[V_{\mathrm{Mo}}^{x}\right]$ as functions of ${\left(P_{S}\right)}_{\min}^{7}$. Based on the charge neutrality conditions discussed above, the evolution of vacancy concentration with varying S vapor pressure is determined. The approximate solution to the equations in Table 1 is illustrated in Figure 2.

At lower values of ${\left(P_{S}\right)}_{\min}^{7}$, the approximation $n=[V_{S}^{+}]$ is valid, as in regions of low S vapor pressure, singly charged S vacancies contribute electrons to the conduction band. With increasing S partial pressure, there exists a regime where charged defect pairs dominate, $\left[V_{S}^{+}\right]=\left[V_{Mo}^{-}\right]$. As the S partial pressure increases further, a regime emerges where the hole concentration is governed by charged Mo vacancies, and the neutrality condition $p=[V_{Mo}^{-}]$ becomes applicable.

### 3.2. Evolution of Film Morphology, Structure and Composition with Varying S Vapor Pressure

The morphology of CVD-grown ${\mathrm{MoS}}_{2}$ is dependent on the temperature, the precursor quantity, and the gas flow rate [18,33]. These factors influence the edge termination, which are the atoms at the outermost edges of the crystal. When the edges are terminated by alternating Mo and S atoms, it results in zigzag edges with Mo zigzag (Mo-zz) and S zigzag (S-zz) edge terminations. The growth rate of different crystal facets, determined by their edge free energy, dictates the shape and edge composition of the ${\mathrm{MoS}}_{2}$ crystal [34]. Low-energy facets grow more slowly, eventually becoming the dominant faces compared to faster-growing ones.

#### 3.2.1. Variations in Film Morphology with Increasing S Pressure

**Figure 3:**
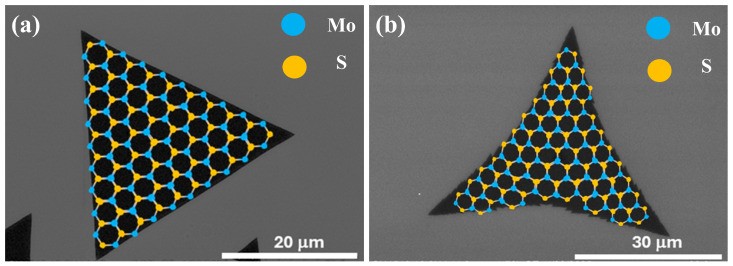
Schematic illustration of edge terminations in ${\mathrm{MoS}}_{2}$ and their influence on morphology. (**a**) Mo-zz edge termination resulting in the formation of well-defined triangular domains; (**b**) S-zz edge termination leading to three-pointed star-like domains.

In a review by Zimeng Ye et al. [35], the authors report that fluctuations in the flux ratio of Mo to S sources can alter the chemical composition of the terminal edges in ${\mathrm{MoS}}_{2}$, thereby influencing the resulting film morphology. A perfect triangular film results from Mo-zz edge termination (shown in Figure 3a) when the Mo:S ratio is at its optimum. An excess of S in the chamber leads to S-zz edge termination, which is accompanied by a structural transformation (shown in Figure 3b).

**Figure 4:**
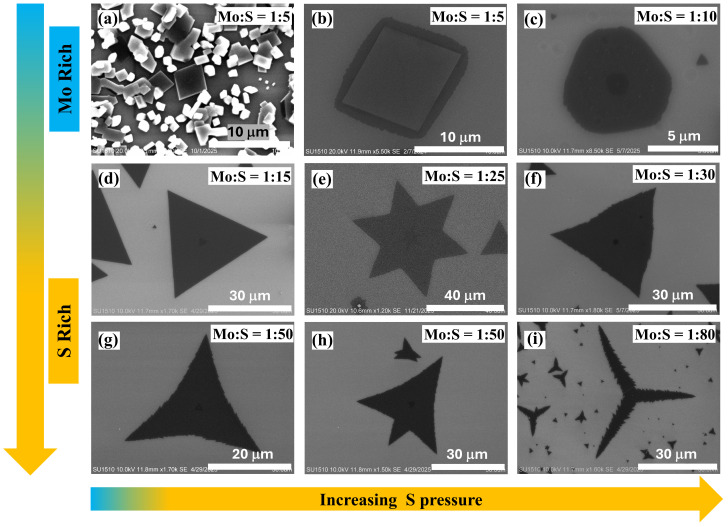
SEM images of ${\mathrm{MoS}}_{2}$ films grown under varying S vapor pressures. The arrows indicate increasing S pressure, caused by increasing the S precursor mass, while holding the Mo mass fixed. (**a**,**b**) are bulk crystals of ${\mathrm{MoO}}_{x}S_{2-x}$ grown under Mo-rich conditions with Mo:S mass ratio of 1:5; (**c**) ${\mathrm{MoS}}_{2}$ monolayers grown with Mo:S ratio of 1:10; (**d**) triangular monolayer ${\mathrm{MoS}}_{2}$ films grown under optimal conditions, with Mo:S mass ratio of 1:15; (**e**–**i**) ${\mathrm{MoS}}_{2}$ films grown with increasing S content in the growth chamber, demonstrating a transition from triangular shapes (monolayers) to dendritic-branched morphologies (multilayers).

**Figure 5:**
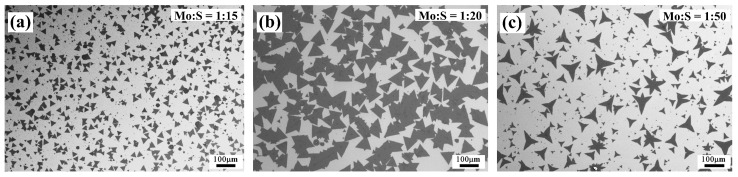
SEM images showing large-area coverage of ${\mathrm{MoS}}_{2}$ grown under increasing S vapor pressure. (**a**) Growth under optimum conditions (Mo:S = 1:15) shows lower areal coverage of triangular films; (**b**) higher areal coverage for films grown with moderately increased S pressure (Mo:S = 1:20); (**c**) film grown under S-rich conditions (Mo:S = 1:50).

These findings were validated by synthesizing ${\mathrm{MoS}}_{2}$ films under varying S pressures to investigate S pressure-dependent structural changes. An insufficient S supply in the growth chamber (Mo-rich conditions, when the Mo:S ratio is 1:5 or lower) favored the formation of molybdenum oxysulfides, ${\mathrm{MoO}}_{x}S_{2-x}$, resulting in sheet-like rectangular, hexagonal, and several truncated morphologies (Figure 4a,b). In this Mo-rich region, a small increase in S pressure (Mo:S = 1:10) results in hexagonal ${\mathrm{MoS}}_{2}$ (Figure 4c), where the number of Mo-zz and S-zz edges are balanced [33]. A well-defined monolayer triangle (Figure 4d) is obtained at a Mo:S precursor molar ratio of 1:15, which we identify as the optimum growth condition in this study. As the S concentration increases, well-formed six 60°-oriented nuclei develop, yielding distinct six-pointed star-shaped flakes (Figure 4e) [36]. This transition is attributed to the enhanced nucleation density at higher S levels, promoting the coalescence of individual triangular domains into symmetric six-pointed stars. Increasing the S vapor pressure alters the edge growth kinetics: Mo-zz edges advance more rapidly, while S-zz edges grow more slowly and irregularly, resulting in distorted domain shapes. This growth behavior produces three-pointed star-like morphologies that curve instead of forming equilateral triangles, resulting in the shapes as shown in Figure 4f–h. With further increase in S vapor pressure, the shift from edge-limited growth to a kinetically controlled regime explains the emergence of dendritic morphologies (Figure 4i). In this regime, kinetics dominate over thermodynamics, favoring interlayer mass transport and thereby promotes vertical stacking in addition to lateral monolayer expansion [37,38]. The resulting dendrites exhibit symmetric, star-like morphologies, with sub-branches forming at fixed angles (60°) relative to the main branches, reflecting the crystallographic orientation of the underlying monolayer domain [38,39]. With increasing S pressure, the areal density of the ${\mathrm{MoS}}_{2}$ film on the Si/SiO_2_ substrate changes along with the film morphology. Figure 5 shows the areal coverage for films grown under increasing S pressures. At low S pressures, close to $P_{\mathrm{optimum}}$ (Mo:S = 1:15), the side length of the triangular flakes is about 30 μm and their areal coverage on the Si/SiO_2_ substrate is estimated to be about 30% (Figure 5a). As the S pressure increases (Mo:S = 1:20), the areal density increases to cover about 70% of the substrate surface, and the triangular domain size increases to hundreds of microns (Figure 5b). This is attributed to the higher density of nucleation sites. With further increase in the S pressure to $P_{S-\mathrm{rich}}$ (Mo:S = 1:50), there is a reduction in the areal density and formation of three-pointed stars of different sizes (Figure 5c). Thus, precise tuning of the Mo:S precursor ratio enables better control over the shape and areal coverage of ${\mathrm{MoS}}_{2}$ monolayers.

#### 3.2.2. Structural and Compositional Characterization of ${\mathrm{MoS}}_{2}$ Films Grown Under Varying S Pressure

The results discussed in this section correspond to (i) films grown under optimum Mo:S precursor conditions, which yield triangular domains as shown in Figure 4d; and (ii) films grown under high S pressure, which exhibit the morphology presented in Figure 4g.

**Figure 6:**
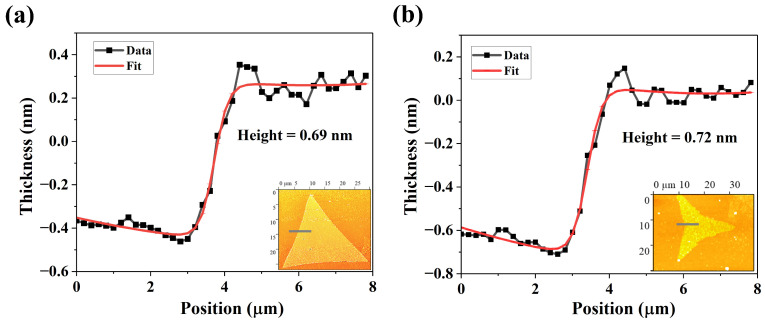
(**a**) The height profile of a ${\mathrm{MoS}}_{2}$ monolayer film (growth condition corresponding to morphology of Figure 4d) with a thickness of 0.69 nm. The inset is the top-view AFM image with its topographic cross-sectional profile measured across the solid gray line. (**b**) The height profile of a ${\mathrm{MoS}}_{2}$ monolayer film (growth condition corresponding to morphology of Figure 4g) of thickness 0.72 nm. The inset is the AFM view of the film, with its topographic cross-sectional profile measured across the solid gray line.

The AFM image of the film shown in Figure 6 reveals a step height of approximately 0.7 nm from the substrate to the edge of the film, which is consistent with the thickness of a monolayer ${\mathrm{MoS}}_{2}$ [20]. No change in film thickness was observed for samples grown under different S pressures, indicating that variations in S pressure primarily influences the defect concentration and morphology of the films rather than their vertical growth.

**Figure 7:**
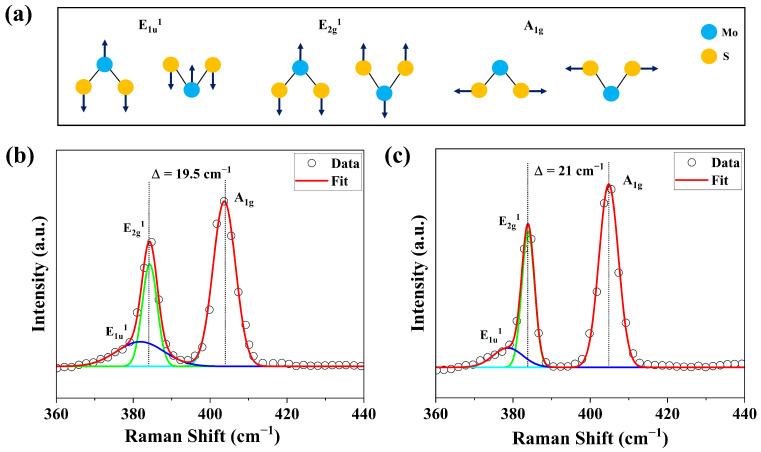
(**a**) Atomic displacement vectors of the $E_{1u}^{1}$, $E_{2g}^{1}$, and $A_{1g}$ modes of ${\mathrm{MoS}}_{2}$. (**b**) Raman spectrum of ${\mathrm{MoS}}_{2}$ films grown under optimum conditions. (**c**) Raman spectrum of ${\mathrm{MoS}}_{2}$ films grown under S-rich condition.

To further investigate the effects of varying S pressure on the ${\mathrm{MoS}}_{2}$ film, Raman spectra was recorded for films grown under optimum and high S pressure. Conventional Raman spectroscopy studies in most reports on ${\mathrm{MoS}}_{2}$ films describe two characteristic first-order Raman-active modes, the in-plane vibration mode ($E_{2g}^{1}$) and the out-of-plane vibration mode ($A_{1g}$), illustrated schematically in Figure 7a [40]. In addition, there are degenerate lattice modes like the $E_{1u}^{1}$ that are IR active. The Raman spectra of ${\mathrm{MoS}}_{2}$ films grown under optimum S pressure and under S-rich conditions are shown in Figure 7b,c.

The Raman spectrum shown in Figure 7b exhibits three characteristic peaks corresponding to the Raman-active modes of hexagonal ${\mathrm{MoS}}_{2}$. The in-plane vibration mode $E_{1u}^{1}$ and $E_{2g}^{1}$ appears at 381.8 ${\mathrm{cm}}^{-1}$ and 384.2 ${\mathrm{cm}}^{-1}$, respectively, for films grown under optimum conditions. These are the in-plane Raman-active vibration modes in hexagonal ${\mathrm{MoS}}_{2}$ monolayers, and their frequencies are highly sensitive to strain and interlayer coupling. The out-of-plane vibration mode ($A_{1g}$) is observed at 403.7 ${\mathrm{cm}}^{-1}$ [41] and corresponds to vibrations of the two S layers moving in opposite directions (Figure 7a).

For films grown under sulfur-rich conditions (Figure 7c), the redshift of the $E_{1u}^{1}$ and $E_{2g}^{1}$ peaks to 378.7 ${\mathrm{cm}}^{-1}$ and 383.9 ${\mathrm{cm}}^{-1}$, respectively, is attributed to the incorporation of excess sulfur, which perturbs the in-plane Mo-S bond network and modifies the local strain. In contrast, the blueshift of the $A_{1g}$ mode to 404.8 ${\mathrm{cm}}^{-1}$ is likely due to phonon confinement effects [42]. These spectral shifts are consistent with the earlier observation that S pressure does not change film thickness but strongly influences vacancy defect concentration and lattice disorder. The difference in peak positions (Δ) of the $E_{2g}^{1}$ and $A_{1g}$ modes for films grown under optimum and S-rich conditions is 19.5 ${\mathrm{cm}}^{-1}$ and 21.0 ${\mathrm{cm}}^{-1}$, respectively, which falls within the range typically associated with monolayer ${\mathrm{MoS}}_{2}$ [43,44,45]. Raman analysis revealed a reduction in the full width at the half-maximum (FWHM) of the $E_{2g}^{1}$ and $A_{1g}$ modes with increasing S pressure, indicative of decreased film disorder. Under optimum conditions, the FWHM values were 3.9 and 5.9 ${\mathrm{cm}}^{-1}$ for the $E_{2g}^{1}$ and $A_{1g}$ modes, respectively, decreasing to 3.3 and 5.0 ${\mathrm{cm}}^{-1}$ under S-rich conditions.

### 3.3. Electrical Properties of MoS_2_ Films Grown Under Varying S Pressure

The electronic structure of ${\mathrm{MoS}}_{2}$ monolayers is strongly affected by S vacancies, which introduce defect states within the bandgap. The energies and intensities of these states vary with the vacancy concentration and their charged states. It influences carrier scattering, trap-assisted transport, and, ultimately, the electrical performance of the films. To quantitatively evaluate the electrical performance of the ${\mathrm{MoS}}_{2}$ films grown under optimum and S-rich conditions, FET devices were fabricated and four parameters were analyzed for comparison: carrier concentration ($n_{2D}$), field-effect mobility ($\mu_{\mathrm{FE}}$), current switching ratio ($I_{\mathrm{ON}}/I_{\mathrm{OFF}}$), and threshold voltage ($V_{T}$). Both devices exhibited typical FET characteristics of n-type semiconductors, with the drain–source current ($I_{\mathrm{ds}}$) increasing under positive gate bias ($V_{\mathrm{gs}}$), confirming electron-dominated conduction.

**Figure 8:**
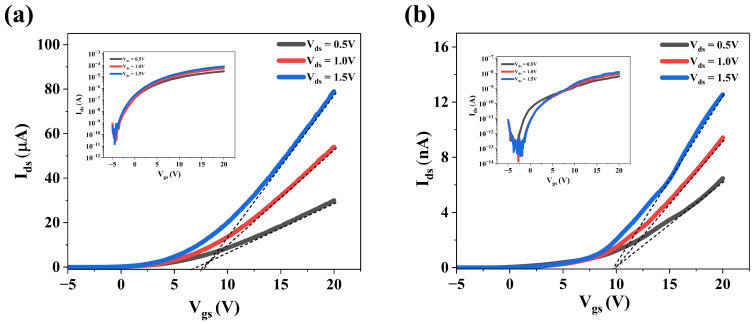
$I_{\mathrm{ds}}-V_{\mathrm{gs}}$ measurement for drain–source voltages 0.5 V, 1.0 V, and 1.5 V, with inset showing the semi-log plot of (**a**) ${\mathrm{MoS}}_{2}$ grown at optimum conditions; (**b**) ${\mathrm{MoS}}_{2}$ grown in S-rich condition.The dashed line in (**a**,**b**) is the linear extrapolation of the $I_{\mathrm{ds}}-V_{\mathrm{gs}}$ characteristics at maximum slope.

The transfer characteristics of the monolayer film grown under optimum and S-rich conditions (morphology shown in Figure 4d,g) are shown in Figure 8a,b, respectively, in both logarithmic (inset) and linear scales. The plots show the variation in I_ds_ with V_gs_ at three different drain–source biases (V_ds_). On the linear scale, I_ds_ shows a linear dependence on V_gs_ in the range of 15–20 V. The dashed line represents the best linear least-squares fit within this region, and the threshold voltage is determined by extrapolating this fit to the point of zero drain current density [20,46,47].

The average mobility in the linear region was subsequently determined according to the following equation [20]:

(5) $$\mu_{\mathrm{FE}}=\frac{L}{WC_{\mathrm{ox}}V_{\mathrm{ds}}}\left(\frac{dI_{\mathrm{ds}}}{dV_{\mathrm{gs}}}\right)$$

where L≈3.0 μm, W≈50 μm, $C_{\mathrm{ox}}=1.23\times 10^{-4}\;F/m^{2}$ is the gate capacitance per unit area for 285 nm thick ${\mathrm{SiO}}_{2}$.

The carrier concentration was estimated from the back-gate voltage using the relation [48]

(6) $$n_{2D}=\frac{C_{\mathrm{ox}}}{e}\left(V_{\mathrm{bg}}-V_{T}\right)$$

Table 2 shows a comparison of the four basic FET parameters for devices fabricated with films grown under optimum and S-rich conditions (schematic shown in Figure 1a). It has been reported that intrinsic defects in ${\mathrm{MoS}}_{2}$ dominate the Au/${\mathrm{MoS}}_{2}$ contact resistance and lead to a low Schottky barrier that is largely independent of the metal’s work function [49]. Experimental evidence shows that metal contacts such as Ti, Pd, and Au with ${\mathrm{MoS}}_{2}$ exhibit low electron Schottky barrier heights despite significant differences in their respective work functions. The sulfur vacancy defects are believed to cause Fermi level pinning approximately 0.2–0.4 eV below the conduction band edge, which is the most probable mechanism responsible for the reduction in Schottky barrier height observed for high work function metals in contact with ${\mathrm{MoS}}_{2}$. For the films under study, the contact resistance ($R_{c}$) was extracted from four-probe measurements, where the total resistance ($R_{t}$) comprises the ${\mathrm{MoS}}_{2}$ film resistance ($R_{\mathrm{film}}$) and twice the contact resistance. The four contacts in the device were 3.0 μm apart and the film under study had an edge length of about 30.0 μm (schematic shown in Figure 1b). For $R_{\mathrm{film}}=0.18$ MΩ, $R_{c}$ was estimated to be 8.5 kΩ, accounting for approximately 5% of the total device resistance.

As shown in Table 2, the carrier concentration ($n_{2D}$) in films grown under both optimum and sulfur-rich conditions are comparable, and varies from 9.6 $\times \;10^{11}$ ${\mathrm{cm}}^{-2}$ to 7.5 $\times \;10^{11}$ ${\mathrm{cm}}^{-2}$, respectively. A correlation exists between vacancy concentration and the experimentally determined carrier density for films grown under optimum and S-rich conditions, in agreement with the modeling results shown in Figure 2. There are reports that show that, in addition to vacancy concentration, the planar arrangement of defects will also contribute to the overall charge transport [50].

The reduction in $\mu_{\mathrm{FE}}$ and the current switching ratio $I_{\mathrm{ON}}/I_{\mathrm{OFF}}$ is explained on the basis of defects that contribute to scattering. As shown in Figure 2, films grown under both optimum and sulfur-rich conditions (P_optimum_ and P_S-rich_) exhibit variations in stoichiometry arising from differences in the relative concentrations of neutral $\left(\left[V_{S}^{x}\right]\right)$ and positively charged sulfur vacancies $\left(\left[V_{S}^{+}\right]\right)$. For films grown at P_optimum_, $\left[V_{S}^{x}\right]$ dominates over $\left[V_{S}^{+}\right]$ by approximately two orders of magnitude. In contrast, at P_S-rich_, the concentration of the charged vacancies $\left[V_{S}^{+}\right]$ become comparable to that of $\left[V_{S}^{x}\right]$. Moreover, under an applied electric field, $\left[V_{S}^{x}\right]$ can transform into $\left[V_{S}^{+}\right]$ [51], thereby increasing the ionized vacancy concentration and its distribution in the crystal lattice. These charged defect sites serve as impurity scattering centers within the ${\mathrm{MoS}}_{2}$ channel, where electrons are scattered by Coulombic interactions, thereby impeding carrier transport, reducing $\mu_{\mathrm{FE}}$, and degrading the overall device performance [52,53,54]. The results of the electrical characterization studies suggest that intrinsic defects, especially charged vacancy defects, are responsible for the deterioration of the ${\mathrm{MoS}}_{2}$-based FET device performance. In previous studies, the average two-dimensional carrier density ($n_{2D}$) in the channels of monolayer ${\mathrm{MoS}}_{2}$ FETs, estimated using similar analytical models of the transfer characteristics, has been reported to vary from approximately 1.4 $\times \;10^{11}$ ${\mathrm{cm}}^{-2}$ [55] to 2.8 $\times \;10^{12}$ ${\mathrm{cm}}^{-2}$ [56] to as high as 1.8 $\times \;10^{13}$ ${\mathrm{cm}}^{-2}$ [57]. It is noted that the average carrier concentration will be determined by several factors that include defect fluctuations in the monolayer, the film/substrate interfaces, local structural variations like wrinkles in the film, and surface contamination that could come from the PMMA-assisted lift-off technique.

## 4. Conclusions

The morphology, structural, optical, and electrical properties of monolayer ${\mathrm{MoS}}_{2}$ films grown by CVD were found to be strongly influenced by the precursor Mo:S vapor flux ratio. A thermodynamic model was developed to analyze the dependency of vacancy defect concentrations on sulfur vapor pressure. The study identified a specific range of precursor ratios suitable for achieving monolayer ${\mathrm{MoS}}_{2}$ growth. At low sulfur pressures, molybdenum oxysulfide phases were favored, leading to bulk crystal formation. With increasing sulfur concentration, the film morphology transitioned from well-defined triangular domains to distorted, non-equilateral structures, and ultimately to dendritic growth at higher S flux. An optimum Mo:S ratio was determined, enabling the large-area growth of triangular monolayer domains.

Electrical measurements revealed that devices fabricated from films grown at the optimum sulfur pressure ($P_{\mathrm{optimum}}$) exhibited superior electronic properties, with carrier concentrations of the order of $10^{11}$ ${\mathrm{cm}}^{-2}$ and higher field-effect mobility, making them favorable for device applications. In contrast, films grown under sulfur-rich conditions ($P_{S\text{-}\mathrm{rich}}$) showed degraded field-effect mobility and current on/off ratios, which are attributed to increased impurity scattering caused by the higher concentration of charged sulfur vacancies. These findings provide important insights into the correlation between growth parameters, defect chemistry, and electronic performance, offering valuable guidelines for tailoring the properties of two-dimensional semiconductors through controlled vapor-phase synthesis.

## Figures and Tables

**Table 1:** Reaction equations and corresponding equilibrium constants for vacancy formation.

Reaction Equations for Formation of Vacancies	Equilibrium Constant Equations (Mass–Action Relations)
2 MoO_3(g)_ + 7 S_(g)_ ⇌ 2 MoS_2__(s)_ + 3 SO_2__(g)_	K_*f*_ = ${P_{\mathrm{MoS}2}}^{2}$ · ${P_{S}}^{7}$
7 S(v) ⇌ ${S_{S}}^{x}$ + ${V_{\mathrm{Mo}}}^{x}$	K_*v*_ = [${V_{\mathrm{Mo}}}^{x}$]/${P_{S}}^{7}$
${V_{\mathrm{Mo}}}^{x}$ + ${V_{S}}^{x}$ ⇌ 0	${K_{S}}^{x}$ = [${V_{\mathrm{Mo}}}^{x}$][${V_{S}}^{x}$]
${V_{S}}^{x}$ ⇌ ${V_{S}}^{+}$ + e^−^	K_*d*_ = [${V_{S}}^{+}$] n/[${V_{S}}^{x}$]
${V_{\mathrm{Mo}}}^{x}$ ⇌ ${V_{\mathrm{Mo}}}^{-}$ + h^+^	K_*a*_ = [${V_{\mathrm{Mo}}}^{-}$] p/[${V_{\mathrm{Mo}}}^{x}$]
0 ⇌ n + p	K_*i*_ = n · p

**Table 2:** Comparison of device parameters under optimum and S-rich growth conditions.

Parameter	Optimum	S-Rich
$n_{2D}$ (${\mathrm{cm}}^{-2}$)	$9.6\times 10^{11}$	$7.5\times 10^{11}$
$\mu_{\mathrm{FE}}$ (${\mathrm{cm}}^{2}$/Vs)	20.4	13.1
$V_{T}$ at $V_{\mathrm{ds}}=1$ V (V)	7.5	9.7
$I_{\mathrm{ON}}/I_{\mathrm{OFF}}$	$5.5\times 10^{6}$	$7.0\times 10^{5}$

## Data Availability

The original contributions presented in this study are included in the article. Further inquiries can be directed to the corresponding author.

## References

[1] He Z., Que W. (2016). Molybdenum disulfide nanomaterials: Structures, properties, synthesis and recent progress on hydrogen evolution reaction. Appl. Mater. Today.

[2] Nalwa H.S. (2020). A review of molybdenum disulfide (MoS_2_) based photodetectors: From ultra-broadband, self-powered to flexible devices. RSC Adv..

[3] Xiao D., Liu G.-B., Feng W., Xu X., Yao W. (2012). Coupled spin and valley physics in monolayers of MoS_2_ and other group-VI dichalcogenides. Phys. Rev. Lett..

[4] Mak K.F., Lee C., Hone J., Shan J., Heinz T.F. (2010). Atomically thin MoS_2_: A new direct-gap semiconductor. Phys. Rev. Lett..

[5] Tang Q., Jiang D.-E. (2015). Stabilization and band-gap tuning of the 1T-MoS_2_ monolayer by covalent functionalization. Chem. Mater..

[6] Radisavljevic B., Whitwick M.B., Kis A. (2011). Integrated circuits and logic operations based on single-layer MoS_2_. ACS Nano.

[7] Wang H., Yu L., Lee Y.-H., Shi Y., Hsu A., Chin M.L., Li L.-J., Dubey M., Kong J., Palacios T. (2012). Integrated circuits based on bilayer MoS_2_ transistors. Nano Lett..

[8] Nur R., Tsuchiya T., Toprasertpong K., Terabe K., Takagi S., Takenaka M. (2020). High responsivity in MoS_2_ phototransistors based on charge trapping HfO_2_ dielectrics. Commun. Mater..

[9] Liu K., Wang X., Su H., Chen X., Wang D., Guo J., Shao L., Bao W., Chen H. (2022). Large-scale MoS_2_ pixel array for imaging sensor. Nanomaterials.

[10] Park J., Mun J., Shin J.-S., Kang S.-W. (2018). Highly sensitive two-dimensional MoS_2_ gas sensor decorated with Pt nanoparticles. R. Soc. Open Sci..

[11] Chen Y., Vicente N., Pham T., Mulchandani A. (2025). Biological sensing using vertical MoS_2_-graphene heterostructure-based field-effect transistor biosensors. Biosensors.

[12] Tsai M.-Y., Tarasov A., Hesabi Z.R., Taghinejad H., Campbell P.M., Joiner C.A., Adibi A., Vogel E.M. (2015). Flexible MoS_2_ field-effect transistors for gate-tunable piezoresistive strain sensors. ACS Appl. Mater. Interfaces.

[13] Hao Y., Zhang S., Fan C., Liu J., Hao S., Lu X., Zhou J., Qiu M., Li J., Hao G. (2025). Te nanomesh-monolayer WSe_2_ vertical van der Waals heterostructure for high-performance photodetector. Appl. Phys. Lett..

[14] Hao S., Hao Y., Li J., Wang K., Fan C., Zhang S., Wei Y., Hao G. (2024). Controllable growth of two-dimensional wrinkled WSe_2_ nanostructures via chemical vapor deposition based on thermal mismatch strategy. Appl. Phys. Lett..

[15] Schmidt H., Wang S., Chu L., Toh M., Kumar R., Zhao W., Castro Neto A.H., Martin J., Adam S., Özyilmaz B. (2014). Transport properties of monolayer MoS_2_ grown by chemical vapor deposition. Nano Lett..

[16] Zhou W., Zou X., Najmaei S., Liu Z., Shi Y., Kong J., Lou J., Ajayan P.M., Yakobson B.I., Idrobo J.-C. (2013). Intrinsic structural defects in monolayer molybdenum disulfide. Nano Lett..

[17] Macchione M.A., Mendoza-Cruz R., Bazán-Diaz L., Velázquez-Salazar J.J., Santiago U., Arellano-Jiménez M.J., Perez J.F., José-Yacamán M., Samaniego-Benitez J.E. (2020). Electron microscopy study of the carbon-induced 2H–3R–1T phase transition of MoS_2_. New J. Chem..

[18] Yang S.Y., Shim G.W., Seo S.-B., Choi S.-Y. (2017). Effective shape-controlled growth of monolayer MoS_2_ flakes by powder-based chemical vapor deposition. Nano Res..

[19] Feng L.-P., Su J., Liu Z.-T. (2015). Effect of vacancies in monolayer MoS_2_ on electronic properties of Mo–MoS_2_ contacts. RSC Adv..

[20] Jiang Y., Lin Y., Littler C., Syllaios A.J., Neogi A., Philipose U. (2021). Analyzing growth kinematics and fractal dimensions of molybdenum disulfide films. Nanotechnology.

[21] Lee Y.-H., Zhang X.-Q., Zhang W., Chang M.-T., Lin C.-T., Chang K.-D., Yu Y.-C., Wang J.T.-W., Chang C.-S., Li L.-J. (2012). Synthesis of large-area MoS_2_ atomic layers with chemical vapor deposition. arXiv.

[22] Weber T., Muijsers J.C., Van Wolput J.H.M.C., Verhagen C.P.J., Niemantsverdriet J.W. (1996). Basic reaction steps in the sulfidation of crystalline MoO_3_ to MoS_2_, as studied by X-ray photoelectron and infrared emission spectroscopy. J. Phys. Chem..

[23] Farmer G., Abraham J., Littler C., Syllaios A.J., Philipose U. (2022). Growth of highly-ordered metal nanoparticle arrays in the dimpled pores of an anodic aluminum oxide template. Nanomaterials.

[24] Pondick J.V., Woods J.M., Xing J., Zhou Y., Cha J.J. (2018). Stepwise sulfurization from MoO_3_ to MoS_2_ via chemical vapor deposition. ACS Appl. Nano Mater..

[25] Wu S., Huang C., Aivazian G., Ross J.S., Cobden D.H., Xu X. (2013). Vapor–solid growth of high optical quality MoS_2_ monolayers with near-unity valley polarization. ACS Nano.

[26] Ansh A., Patbhaje U., Kumar J., Meersha A., Shrivastava M. (2023). Origin of electrically induced defects in monolayer MoS_2_ grown by chemical vapor deposition. Commun. Mater..

[27] Reshchikov M.A., Morkoç H. (2005). Luminescence properties of defects in GaN. J. Appl. Phys..

[28] Philipose U., Sapkota G. (2013). Defect formation in InSb nanowires and its effect on stoichiometry and carrier transport. J. Nanopart. Res..

[29] Smidstrup S., Stokbro K., Blom A., Markussen T., Wellendorff J., Schneider J., Gunst T., Verstichel B., Khomyakov P.A., Vej-Hansen U.G. (2020). QuantumATK: An integrated platform of electronic and atomic-scale modelling tools. J. Phys. Condens. Matter.

[30] Smidstrup S., Stradi D., Wellendorff J., Khomyakov P.A., Vej-Hansen U.G., Lee M.-E., Ghosh T., Jónsson E., Jónsson H., Stokbro K. (2017). First-principles Green’s-function method for surface calculations: A pseudopotential localized basis set approach. Phys. Rev. B.

[31] Chase M.W. (1998). NIST-JANAF thermochemical tables. J. Phys. Chem. Ref. Data.

[32] Chadi D.J. (1994). The problem of doping in II-VI semiconductors. Annu. Rev. Mater. Sci..

[33] Wang S., Rong Y., Fan Y., Pacios M., Bhaskaran H., He K., Warner J.H. (2014). Shape evolution of monolayer MoS_2_ crystals grown by chemical vapor deposition. Chem. Mater..

[34] Chen Q., Li H., Xu W., Wang S., Sawada H., Allen C.S., Kirkland A.I., Grossman J.C., Warner J.H. (2017). Atomically flat zigzag edges in monolayer MoS_2_ by thermal annealing. Nano Lett..

[35] Ye Z., Tan C., Huang X., Ouyang Y., Yang L., Wang Z., Dong M. (2023). Emerging MoS_2_ wafer-scale technique for integrated circuits. Nano-Micro Lett..

[36] Prasad R.K., Singh D.K. (2023). Continuous large area monolayered molybdenum disulfide growth using atmospheric pressure chemical vapor deposition. ACS Omega.

[37] Xu W., Li S., Zhou S., Lee J.K., Wang S., Sarwat S.G., Wang X., Bhaskaran H., Pasta M., Warner J.H. (2018). Large dendritic monolayer MoS_2_ grown by atmospheric pressure chemical vapor deposition for electrocatalysis. ACS Appl. Mater. Interfaces.

[38] Tyagi M., Tiwari A.A., Dey S., Sahdev D. (2024). Chemical vapor deposition growth of large-area molybdenum disulphide (MoS_2_) dendrites. Nano-Struct. Nano-Objects.

[39] Liu Z., Amani M., Najmaei S., Xu Q., Zou X., Zhou W., Yu T., Qiu C., Birdwell A.G., Crowne F.J. (2014). Strain and structure heterogeneity in MoS_2_ atomic layers grown by chemical vapour deposition. Nat. Commun..

[40] Ye M., Winslow D., Zhang D., Pandey R., Yap Y.K. (2015). Recent advancement on the optical properties of two-dimensional molybdenum disulfide (MoS_2_) thin films. Photonics.

[41] Chakraborty B., Matte H.S.S.R., Sood A.K., Rao C.N.R. (2013). Layer-dependent resonant Raman scattering of a few layer MoS_2_. J. Raman Spectrosc..

[42] Isherwood L.H., Hennighausen Z., Son S.-K., Spencer B.F., Wady P.T., Shubeita S.M., Kar S., Casiraghi C., Baidak A. (2020). The influence of crystal thickness and interlayer interactions on the properties of heavy ion irradiated MoS_2_. 2D Mater..

[43] George A., Neumann C., Kaiser D., Mupparapu R., Lehnert T., Hübner U., Tang Z., Winter A., Kaiser U., Staude I. (2019). Controlled growth of transition metal dichalcogenide monolayers using Knudsen-type effusion cells for the precursors. J. Phys. Mater..

[44] Markeev P.A., Najafidehaghani E., Gan Z., Sotthewes K., George A., Turchanin A., de Jong M.P. (2021). Energy-level alignment at interfaces between transition-metal dichalcogenide monolayers and metal electrodes studied with Kelvin probe force microscopy. J. Phys. Chem. C.

[45] Roy A., Ghosh R., Rai A., Sanne A., Kim K., Movva H.C.P., Dey R., Pramanik T., Chowdhury S., Tutuc E. (2017). Intra-domain periodic defects in monolayer MoS_2_. Appl. Phys. Lett..

[46] Shen T., Li F., Xu L., Zhang Z., Qiu F., Li Z., Qi J. (2020). High mobility monolayer MoS_2_ transistors and its charge transport behaviour under E-beam irradiation. J. Mater. Sci..

[47] Ortiz-Conde A., Sánchez F.J.G., Liou J.J. (2000). On the extraction of threshold voltage, effective channel length and series resistance of MOSFETs. J. Telecommun. Inf. Technol..

[48] Liu X., Hu J., Yue C., Della Fera N., Ling Y., Mao Z., Wei J. (2014). High performance field-effect transistor based on multilayer tungsten disulfide. ACS Nano.

[49] McDonnell S., Addou R., Buie C., Wallace R.M., Hinkle C.L. (2014). Defect-dominated doping and contact resistance in MoS_2_. ACS Nano.

[50] Kuperman Benedik H., Rom N., Caspary Toroker M. (2025). The effect of sulfur vacancy distribution on charge transport across MoS_2_ monolayers: A quantum mechanical study. ACS Mater. Au.

[51] Hu L., Yang J., Wang J., Cheng P., Chua L.O., Zhuge F. (2021). All-optically controlled memristor for optoelectronic neuromorphic computing. Adv. Funct. Mater..

[52] Ferry D.K. (2017). Electron transport in some transition metal di-chalcogenides: MoS_2_ and WS_2_. Semicond. Sci. Technol..

[53] Baugher B.W.H., Churchill H.O.H., Yang Y., Jarillo-Herrero P. (2013). Intrinsic electronic transport properties of high-quality monolayer and bilayer MoS_2_. Nano Lett..

[54] Ghatak S., Pal A.N., Ghosh A. (2011). Nature of electronic states in atomically thin MoS_2_ field-effect transistors. ACS Nano.

[55] Kang D.-H., Kim M.-S., Shim J., Jeon J., Park H.-Y., Jung W.-S., Yu H.-Y., Pang C.-H., Lee S., Park J.-H. (2015). High-performance transition metal dichalcogenide photodetectors enhanced by self-assembled monolayer doping. Adv. Funct. Mater..

[56] Tu H.-W., Shih C.-C., Lin C.-L., Yu M.-Z., Lai J.-J., Luo J.-C., Lin G.-L., Jian W.-B., Watanabe K., Taniguchi T. (2021). High field-effect performance and intrinsic scattering in the two-dimensional MoS_2_ semiconductors. Appl. Surf. Sci..

[57] Chakraborty B., Bera A., Muthu D.V.S., Bhowmick S., Waghmare U.V., Sood A.K. (2012). Symmetry-dependent phonon renormalization in monolayer MoS_2_ transistor. Phys. Rev. B.

